# A Cost-Effective Integrated Methodology for Electromagnetic Actuation via Visual Feedback

**DOI:** 10.3390/s24092760

**Published:** 2024-04-26

**Authors:** Shuwan Chen, Damiano Padovani, Andrea Cioncolini, Angelo Alessandri

**Affiliations:** 1 Department of Mechanical Engineering, Technion-Israel Institute of Technology, Haifa 3200003, Israel; shuwan.chen@campus.technion.ac.il; 2 Department of Mechanical Engineering (Robotics), Guangdong Technion-Israel Institute of Technology, Shantou 515063, China; angelo.alessandri@gtiit.edu.cn; 3 Department of Mechanical Engineering DIME, University of Genoa, 16145 Genoa, Italy

**Keywords:** real-time machine vision, magnetic-driven actuation, closed-loop feedforward control, magnetic microrobots

## Abstract

Electromagnetic actuation can support many fields of technology, such as robotics or biomedical applications. In this context, fully understanding the system behavior and proposing a low-cost package for feedback control is challenging. Modeling the electromagnetic force is particularly tricky because it is a nonlinear function of the actuated object’s position and coil’s current. Measuring in real time the position of the actuated object with the precision required for accurate motion control is also nontrivial. In this study, we propose a novel, cost-effective electromagnetic set-up to achieve position control via visual feedback. We actuated vertically and under different experimental conditions a 10 mm diameter steel ball hanging on a low-stiffness spring, demonstrating good tracking performance (the position error remained within ±0.5 mm, with a negligible phase delay in the best scenarios). The experimental results confirm the feasibility of the proposed set-up, which is characterized by minimum complexity and realized with off-the-shelf and cost-effective components. For these reasons, such a contribution helps to understand and apply electromagnetic actuation even further.

## 1. Introduction

Electromagnetic actuation finds application in many engineering sectors since its driving magnetic field can easily and safely propagate through many materials and reach confined spaces. These unique features make this actuation form well-suited for multiple applications broadly related to robotics, such as electromagnetic actuation for image stabilization [1], minimally invasive microrobots for microsurgery [2,3,4,5,6,7,8], targeted drug delivery inside the human body [9,10], early screening and cancer treatment [11], magnetic levitation (e.g., vertical motion of a ball [12]), and general motion control of ferromagnetic elements (e.g., planar steering of a ferrofluid drop [13]).

It is well-known that electromagnets can only pull ferromagnetic objects without the ability to push them away. Thus, precisely manipulating a ferromagnetic object at a distance from the electromagnet(s) is challenging for multiple reasons. Modeling the electromagnetic force is quite uncertain because it is a nonlinear function of the actuated object position and electromagnet coil current (the magnetic flux and the resulting electromagnet’s pulling force drop quickly with the distance from its surface). Analytical expressions available to predict the electromagnetic force have limited accuracy and are not readily usable for control design due to their complexity, even when referencing idealized scenarios [14,15,16,17]. Moreover, the presence of a ferromagnetic element in the close vicinity of an electromagnet can significantly distort the magnetic flux distribution, an effect not normally incorporated in analytical prediction methods (refer to Section 2.3 for more details). Fully understanding the system behavior is therefore difficult in this context. 

An additional difficulty takes over when the goal becomes achieving closed-loop position control of the actuated element. The object position must be sensed without interfering with its motion. Thus, feasible alternatives for industrial settings use laser sensors [17] or optical tracking [13]. Other expensive techniques exist (e.g., magnetic resonance imaging, computed tomography, ultrasound, or infrared radiation [2]). Still, they are more suitable for biomedical applications, such as magnetic particle imaging used for electromagnetically navigating drug delivery [18]. Laser sensing is costly and has some challenges, such as safety-related issues, because lasers can harm the human eye. Optical tracking can be cost-effective, or at least affordable, depending on the selected camera used for visual feedback (the required frame rate, which is application-driven, plays a significant role). In any case, the latter technique is promising when cost-effectiveness is sought. 

Concerning motion control in industrial applications, the steering of ferromagnetic particles (ferrofluid) was studied in 2D. The proposed form of planar manipulation relied on an array of four electromagnets actuated by feedback control based on visual feedback [19]. The ferrofluid could follow arbitrary trajectories with reasonable accuracy, which was improved using optimal control techniques [13,20]. Despite the remarkable tracking abilities, this approach needs complex control design and is limited to systems with slow dynamics (the feedback loop runs at 15 Hz). Then, an approach with 3D motion capabilities in high-viscosity fluids drove a 1.46 mg magnetic cylinder via eight electromagnets [21]. The magnetic force distribution in the workspace was not precisely known, so the controller involved a proportional-integral-derivative (PID) design whose tuning was not model-based. A physical-based control design is, in fact, very computationally demanding. A black-box approximation based on neural networks was proposed, but the system’s active control was not further investigated [22]. Moreover, the literature on the control of levitating (solid) objects is extensive. Typical techniques are complex due to the strong nonlinearities of magnetic levitating systems. They involve neural networks [17], feedback linearization [12,23], model predictive control [24], or adaptive sliding mode control [25]. These controllers require the measurement of the actuated object’s position with a fast enough sampling time. Precise knowledge of the electromagnetic force is also crucial for control design, a rarely well-defined scenario.

Referring to motion control in the medical field, tiny dimensions are commonplace. The first example concerns a microrobot with a predefined magnetization profile [26]. The time-varying magnetic field imposed with open-loop control around the robot generates different locomotion modes (e.g., walking, crawling, or jumping). Then, a cylindrical magnetic millirobot can navigate in soft tissues because it is attracted to the center of a permanent magnet array [27]; position control is achieved by x-ray imaging and a motion stage that slides and rotates the soft tissues. Three permanent magnets (only one of them is displaced by a stepper motor) enable precise control of another microrobot to perform complex trajectories and deploy cargo [28]. This method leverages open-loop control, unlike the closed-loop technique developed for a pivot walking robot [29]. The latter device employs visual feedback but needs a complex magnetic actuator with three nested electromagnetic Helmholtz coils.

In summary, position control with visual feedback for electromagnetic actuation requires the accurate and contactless measurement of the object position, the accurate prediction of the electromagnetic force, and the implementation of a suitable control strategy to close the loop. Even though electromagnetic actuation is a well-established technology, most of the existing studies are restricted to the actuation of comparatively small metallic objects. The advantage of actuating small objects is that they do not appreciably distort the electromagnetic field. Therefore, existing analytical or numerical prediction methods can be employed to estimate the magnetic flux distribution around the electromagnet and deduce the corresponding magnetic force exerted onto the actuated object. On the other hand, when the actuated object is not small, its presence distorts the electromagnetic field, making analytical prediction methods not applicable. Numerical prediction methods are still of interest; however, their considerable computational burden makes their incorporation into real-time actuation methodologies impractical. Because of the practical difficulties in accurately predicting the magnetic force, the electromagnetic actuation of comparatively large metallic objects remains largely unexplored despite the potential practical relevance of these scenarios in robotics. This gap in the technical literature created the motivation for the present investigation, whose main objective was to realize an electromagnetic actuation set-up with visual feedback capable of controlling in real-time the position of a metallic object large enough to significantly distort the magnetic field generated by the electromagnet. Specifically, the set-up comprises one webcam for position tracking and one electromagnet actuating a 10 mm diameter steel ball hanging on a low-stiffness spring. Notably, the electromagnetic force has been directly measured, thereby eliminating the uncertainties associated with analytical and numerical prediction methods. More often than not, the internal design of commercially available electromagnets is regarded as sensitive information. It is therefore not disclosed by the manufacturers, which precludes the effective use of numerical prediction methods to estimate the magnetic field and the associated magnetic force. An additional advantage of directly measuring the magnetic force is that precise knowledge of the internal design of the electromagnet is not required, hence making the methodology potentially applicable to any commercially available electromagnet. In conclusion, the present set-up demonstrates good tracking performance with an actuated object large enough to significantly distort the electromagnetic field, even though all of the components used to assemble the set-up are off-the-shelf and cost-effective. 

The rest of this paper is organized as follows. After introducing our novel set-up (Section 2.1), we validate the proposed package for optical tracking of the moving object (Section 2.2). Then, we experimentally identify the field distribution produced by the electromagnet used in our study and the electromagnetic force it generates on the actuated object (Section 2.3). We also characterize the friction force of the moving object in different scenarios (Section 2.4) and propose a feedback control law to perform closed-loop position control (Section 2.5). Finally, we report our experimental results collected under different operating conditions (Section 3) and conclude our discussion with critical remarks and suggestions for future developments (Section 4).

## 2. Experimental Set-Up

This section elucidates the novel set-up developed in this study. After providing a general introduction, we will focus on the different subsystems that we have integrated.

### 2.1. Overall System 

We present our experimental set-up in two steps. First, we give a general description of its operation and then detail the selected components. 

#### 2.1.1. System Description

**Figure 1:**
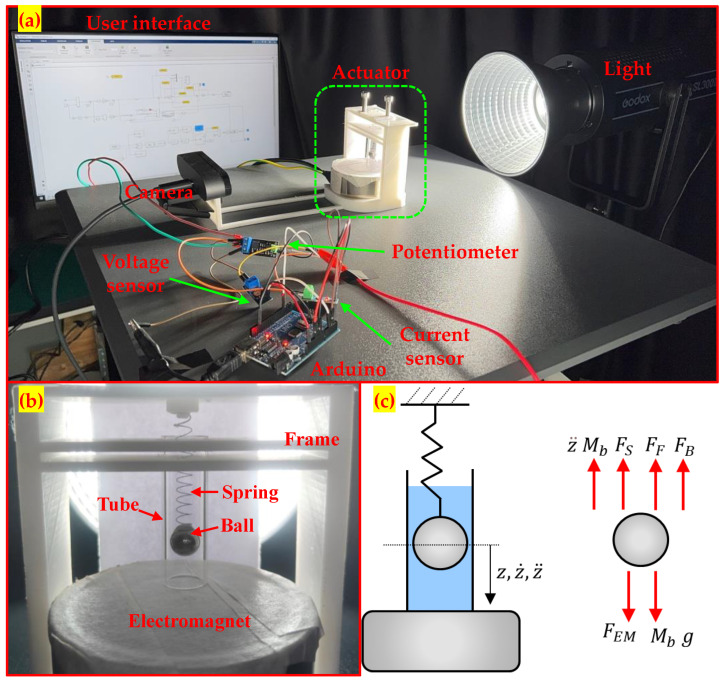
Novel set-up for electromagnetic actuation proposed in this paper: (**a**) system overview, (**b**) zoomed-in view of the actuator and moving object, and (**c**) simplified view of the moving object with its free-body diagram.

Figure 1 shows a simplified representation of the system that we have developed. We aim to control the vertical position of a 10 mm diameter steel ball hanging from a low-stiffness spring connected to a fixed frame. An electromagnet is located below the ball, whose vertical projection intercepting its center ends up on the external radius of the electromagnet core (i.e., the location where the magnetic flux is maximum). We use the axial flux of the electromagnet to lower the ball from its equilibrium position that lies a few millimeters above the electromagnet surface in unactuated conditions; the elastic force of the spring recalls the ball up to the equilibrium position because the electromagnet does not have the capability of lifting the ball. Specifically, a 10 mm diameter ball was selected for use here because it is big enough to significantly distort the magnetic field generated by the present electromagnet, as explained in Section 2.3. Its dimension is representative of the size of magnetically actuated mini robots of interest in biomedical robotics [6,7,11].

The ball is enclosed inside a 13 mm diameter vertical transparent tube filled with fluids characterized by very different viscosity (air, water, or glycol, as explained later), giving opportunities for conducting tests in diverse friction conditions. A camera located laterally and pointing out on one side of the frame measures the vertical ball position. This information is used to track the commanded ball’s position with feedback control acting on the voltage applied to the electromagnet. 

The webcam was positioned as illustrated in Figure 1a. A 3D-printed custom-made plastic support frame (visible in white color in Figure 1a) was realized to hold the electromagnet and the actuated ball, and to provide an anchoring point for the webcam, thereby ensuring that the position of the webcam with respect to the actuated ball remained fixed during the tests. The working distance between the webcam and the actuated ball was fixed at 200 mm, a value identified via trial and error to provide a sharp and well-focused image of the ball with adequate resolution to accurately locate the ball centroid, and was not varied during the tests. Videos of the moving ball were taken with backlighting (the LED light is visible in Figure 1a). Following common practice, a sheet of white paper (visible in Figure 1b) was located behind the actuated ball to provide a uniform background illumination.

The physical governing equations of this set-up are described as follows. The 1D motion of the moving object is captured by Newton’s second law (this assumption of 1D motion holds as confirmed by the experimental evidence available in the attached videos, since the ball always moves vertically without touching the tube):

(1) $$\ddot{z}\;M_{b}=F_{EM}+M_{b}\;g-F_{B}-F_{S}-F_{F},$$

where z is the distance between the electromagnet surface and the ball’s center-of-mass, $M_{b}$ is the ball mass, $F_{EM}$ is the electromagnetic force, g is the acceleration of gravity, $F_{B}$ is the buoyant force, $F_{S}$ is the elastic force of the spring, and $F_{F}$ is the viscous friction force. The buoyant force in Equation (2) recalls the fluid density ρ and fluid volume $V=\pi \;D_{b}^{3}/6$ displaced by the ball with diameter $D_{b}$. The spring force in Equation (3) involves the stiffness constant k, while we model the friction in Equation (4) as a viscous term with a corresponding coefficient b:

(2) $$F_{B}=\rho \;g\;V,$$

(3) $$F_{S}=k\;z+M_{b}\;g-\rho \;g\;V,$$

(4) $$F_{F}=b\;\dot{z}.$$

Modeling the electromagnetic force is challenging because it is a highly nonlinear function of the ball position and the electromagnet’s coil current. This current I is driven by the applied voltage V and depends on the coil resistance R and inductance L, considering that an equivalent R–L circuit can represent the electromagnet:

(5) $$\dot{I}=-\frac{R}{L}I+\frac{1}{L}V.$$

Since the coil resistance remains essentially constant if the temperature does not change significantly, we can express the electromagnetic force as a function of the commanded voltage and ball position (i.e., $F_{EM}=f_{1}\left(z,\;V\right)$) by leveraging the Ohm’s Law (V=R i). We have therefore chosen to fit experimental data as described in Section 2.3 to come up with such an equation of the electromagnetic force.

#### 2.1.2. System Implementation

**Figure 2:**
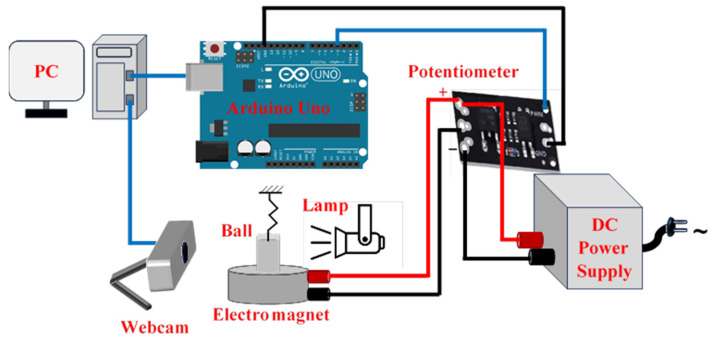
Block diagram showing the structure of the experimental set-up.

We implemented our complete set-up according to the schematic presented in Figure 2. The electromagnetic actuation of the ball is effectuated with a DC-powered lifting solenoid electromagnet KK-P80/38 commercialized by KAKCOM (Yueqing, China). It has a lifting capacity of 120 kg, a maximum operating DC voltage of 24 V, and a maximum current of 0.47 A, corresponding to a maximum power consumption of 11 W. We experimentally characterized the resistance and inductance of the electromagnet using an LCR meter, RS PRO LCR-1701 (Shanghai, China) and found that R= 60.0 Ω and L= 0.6985 H. A 3D-printed frame houses the electromagnet, provides a fixed anchoring point for the spring (its stiffness is k= 0.008 N/mm), and supports the camera used for visual feedback. The latter function is realized with an off-the-shelf Brio C1000e webcam commercialized by Logitech (Lausanne, Switzerland) [30]. Control of the ball motion is achieved by adjusting the voltage applied across the electromagnet. An Arduino 1 board runs the control law that is compiled in Simulink. Then, the control signal leaving the board is directed to a digital potentiometer (Mosfet LR7843, Shenzhen, China) supplied by a 24 V DC electric power supply.

### 2.2. Motion Tracking Methodology

As detailed in the technical specifications [30], the Brio C1000e webcam can be operated with an image resolution of 1920 × 1080 pixels and a recording frequency of 30 fps or 60 fps or with an image resolution of 1280 × 720 pixels and a recording frequency of 30 fps, 60 fps, or 90 fps; the latter corresponds to the maximum recording frequency that this webcam can support. Since a high recording frequency is clearly desirable in motion tracking, the webcam was set to operate at 90 fps with a resolution of 1280 × 720 pixels for the tests documented herein.

Preliminary motion tracking tests indicated that the webcam frame rate did not peak at 90 fps, as expected based on the settings, but instead varied in the range of 85 ± 2 fps. Since modifications to the lighting conditions during the tests had no apparent effect on the webcam frame rate, the minor reduction in the recording frequency from 90 fps to 85 fps was ascribed to the peculiarities of the present set-up, which comprises a close-up view of a relatively small object illuminated with backlighting, and hence does not conform to the typical application of a webcam. Note that increasing the distance between the webcam and the ball to raise the webcam frame rate was not feasible because this would have reduced the spatial resolution, thereby degrading the accuracy of the ball centroid position determination. As shown later, the recording frequency of 85 fps was adequate to track the motion of the ball successfully, and the fluctuation in the frame rate during operation (within ±2 fps) was small enough to be inconsequential on the accuracy of the ball position tracking and subsequent electromagnetic actuation. Therefore, no further action was taken at this stage to raise the frame rate of the webcam, and all motion-tracking videos documented herein were captured at 85 fps. It is worth remembering that the objective of the present study was to develop an integrated methodology for electromagnetic actuation with visual feedback by relying exclusively on cost-effective, off-the-shelf components. Hence, a certain degree of compromise on the performance was inevitable.

The video stream from the webcam was imported in Simulink (version R2021a) using the built-in block ‘From Video Device.’ Simulink ran on a PC DELL Precision 3660 with a 12th Gen Intel(R) Core(TM) i7-12700K processor and 32 GB RAM. The Simulink simulation time step was set at 1/85 s to match the webcam frame rate, namely 85 fps (note that hardware triggering is not supported for the type of webcam employed here). Following standard practice in image analysis, the imported gray-scale frames were binarized and then complemented to isolate one single feature corresponding to the moving ball, whose centroid position was then computed and used to inform the electromagnetic actuation, as explained in Section 2.3. The images were scaled using the ball diameter as reference length, and the spatial resolution was 3.5 pixels/mm. Even though a sub-pixel accuracy on the order of ±0.1 pixels is rather common for centroid location [31], it seems prudent to assume here a more conservative accuracy estimate of ±1 pixel for the ball centroid location, corresponding to about ±300 microns, because the present position tracking methodology was conceived to be cost-effective and hence was not optimized.

**Figure 3:**
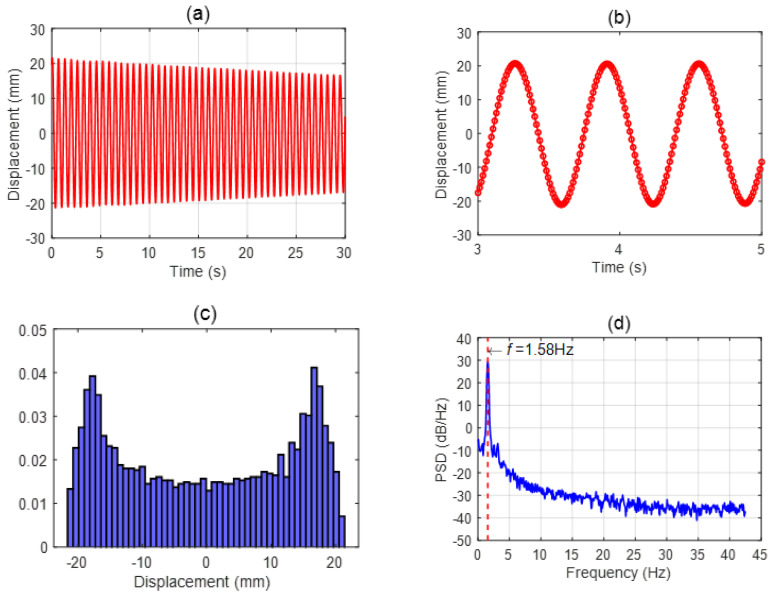
Representative results for motion tracking of the oscillating pendulum: (**a**) displacement time-series, (**b**) detailed view of displacement time-series, (**c**) displacement normalized histogram, (**d**) displacement power spectrum (PSD).

The present motion-tracking methodology was validated with preliminary experiments carried out with an oscillating pendulum. Specifically, the pendulum was realized by suspending the ball from a fixed pivot using a cotton thread of 10 ± 0.1 mm in length. The pendulum was manually displaced from equilibrium, and the subsequent oscillation of the ball was tracked. A representative example is provided in Figure 3, in which the ball displacement time-series and its histogram and power spectrum are presented. As can be noted, the displacement time-series is decaying-periodic. The histogram is bridge-shaped, as expected for the pendulum oscillation, which is a damped periodic motion. The power spectrum presents one dominant peak that closely matches the pendulum oscillation frequency f predicted according to the well-known relation:

(6) $$f=\frac{1}{2\;\pi }\sqrt{\frac{g}{l}}$$

where g is the acceleration of gravity and l is the pendulum length (in the present case, l is 10 mm and hence f is 1.58 Hz). These preliminary pendulum oscillation results validate the present motion tracking set-up and methodology.

### 2.3. Magnetic Flux and Force

The details of the internal design of the electromagnet are unfortunately not divulged by the manufacturer, hence the magnetic properties of the core and the length and number of coil turns of the wire are not available. This precludes the possibility of employing any of the existing analytical or numerical prediction methods to estimate the magnetic flux distribution around the electromagnet, as well as the force exerted by the electromagnet on the actuated ball. As explained later, the actuated ball used here is large enough to interact with the electromagnet, and consequently distort the magnetic flux generated by the electromagnet. Thus, even when the electromagnet’s internal design was known, using numerical prediction methods would require a dedicated calculation for each ball position, which would be impractical for real-time actuation. Therefore, the magnetic flux distribution and the magnetic force on the ball have been directly measured, as detailed in the following paragraphs.

The electromagnet KK-P80/38 selected for use here was introduced above. The attractive force that the electromagnet generates can be varied by adjusting the supply power, which in the present case was produced by modulating the supply voltage from 0 to 24 V. The present electromagnet can generate a maximum attractive force on the ball, which is on the order of ten times the ball weight, hence large enough for the present application.

**Figure 4:**
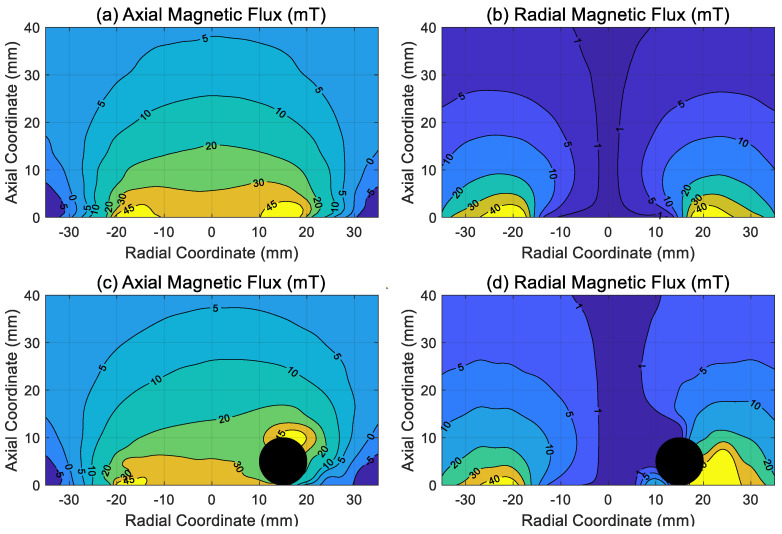
Electromagnet experimental characterization at 24 V supply voltage: (**a**) axial magnetic flux, (**b**) radial magnetic flux, (**c**) axial magnetic flux measured in the presence of the ball, (**d**) radial magnetic flux measured in the presence of the ball. The black circle in panels (**c**,**d**) represents the ball, while the coordinate system is placed on the electromagnet surface with its origin on the geometrical center of the core.

The electromagnet was preliminarily characterized by measuring the magnetic field produced at different supply voltages (the magnetic flux meter employed was the Gaussmeter model GM07 commercialized by Hirst Magnetic Instruments Ltd., Falmouth, UK, www.hirst-magnetics.com, accessed 21 January 2024). Representative results are provided in Figure 4a,b, in which the axial and radial components of the magnetic flux measured at a supply voltage of 24 V are presented as contour plots. Specifically, the radial coordinate is measured along a diameter on the electromagnet surface starting from the geometrical center. In contrast, the axial coordinate is measured perpendicularly to the electromagnet surface, starting from the surface itself. As can be noted in Figure 4a,b, there is a reasonable degree of symmetry in the magnetic field with respect to the axis of the electromagnet (corresponding to the vertical line passing through the origin of the horizontal axis in Figure 4a,b), so that the present electromagnet generates a nearly symmetric magnetic field despite being a cost-effective off-the-shelf component not explicitly designed for sophisticated electromagnetic actuation.

As is well known, the magnetic field generated by an electromagnet is perturbed when magnetizable bodies are present, particularly so when the variation of the magnetic field is significant over a distance comparable to the body dimension. This is clearly the case in the present set-up, where the ball has a diameter of 10 mm and, thus, is large compared to the distance over which the magnetic field varies. As a representative example, the magnetic flux measured in the presence of the ball is provided in Figure 4c,d, where the location of the ball corresponds to the black circle. The perturbation of the magnetic field is evident when comparing the magnetic field measured without the ball (Figure 4a,b) with the corresponding measurements taken when the ball is present (Figure 4c,d). This dramatic modification precludes the possibility of deducing the magnetic force acting on the ball from the magnetic field measurements, such as those documented in Figure 4a,b, which nonetheless retain their value in characterizing the electromagnet performance, notably the degree of symmetry of the magnetic field.

**Figure 5:**
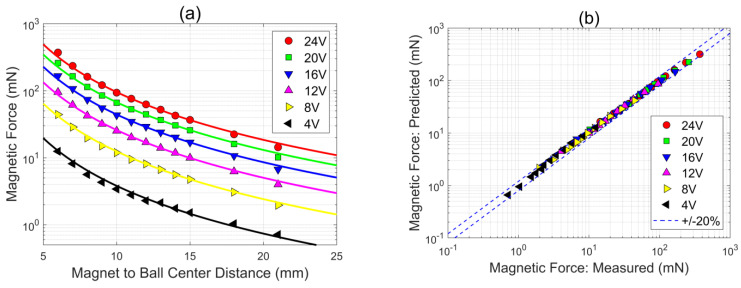
(**a**) Magnetic force measurements at various distances between the electromagnet surface and the ball center for different electromagnet supply voltages (4, 8, 12, 16, 20, and 24 V): the discrete data points are the measured values, while the continuous lines are the predictions of the fitting relation in Equation (7); (**b**) parity plot with comparison between magnetic force predictions from Equation (7) and measurements (the dashed lines are ±20% error bounds).

The magnetic force acting on the ball was measured directly with a force sensor (model ZNLBM-IIX commercialized by BENGBU CHINO SENSOR Co., Ltd., Bengbu, China). To this extent, a custom-made plastic support was 3D printed to connect the ball to the force sensor. Then, the ball was placed at several distances from the electromagnet, and the magnetic force was measured at each distance for different electromagnet supply voltages (4, 8, 12, 16, 20, and 24 V). The results are provided in Figure 5a, where the magnetic force measurements at each electromagnet supply voltage are displayed as functions of the distance between the electromagnet surface and the center of the ball (note that, since the ball diameter is 10 mm, a distance of 5 mm would correspond to the ball sitting on the electromagnet surface). As can be noted, the magnetic force decreases rapidly with increasing distance, and the trends observed at different supply voltages appear qualitatively similar.

For use here, the magnetic force measurements in Figure 5a have been fitted with the following empirical relation:

(7) $$\frac{F_{EM}}{M_{b}\;g}=\left[2.182{\left(\frac{V}{V_{max}}\right)}^{2}+0.2095\left(\frac{V}{V_{max}}\right)\right]\;{\left(\frac{z}{D_{b}}\right)}^{-2.364}$$

where $F_{EM}$ is the magnetic force, $M_{b}$ is the mass of the ball (4.068 g), $D_{b}$ is the diameter of the ball (10.00 mm), g is the acceleration of gravity, V is the electromagnet supply voltage ($V_{max}$ is the maximum supply voltage, equal to 24 V), and z is the distance between the electromagnet surface and the ball center in mm. The functional form of Equation (7), a power law modulated by the supply voltage, is motivated by the trend observed in the data in Figure 5a, and the numerical parameters have been computed with standard least squares regression.

The predictions of the empirical fitting relation in Equation (7) are included in Figure 5a as continuous lines superimposed onto the discrete measurements, while a parity plot with a direct comparison between predictions and measurements is provided in Figure 5b. The empirical relation in Equation (7) fits the present magnetic force measurements quite well, with an accuracy of 2.7% and a bias of 0.6%, which are deemed adequate for the present application. In particular, the accuracy and bias metrics used here are the mean symmetric accuracy and the symmetric signed percentage error [32]. The fitting relation in Equation (7) is also analytically invertible, which simplifies its incorporation into the control methodology, as discussed in Section 2.5.

It is worth noting that the key requirements of the empirical model of the electromagnetic force in Equation (7) are to provide numerically accurate force predictions for the present set-up, while at the same time being analytically simple to facilitate its subsequent incorporation into the control methodology. Clearly, Equation (7) satisfies both of these requirements. This said, even though the applicability of the force relation in Equation (7) is restricted to the present electromagnet/ball combination, the functional form of the correlation can serve as a starting point for different electromagnets and actuated objects. Hence, the practical usefulness of Equation (7) goes beyond the limits of the present study.

### 2.4. Viscous Friction Force 

**Figure 6:**
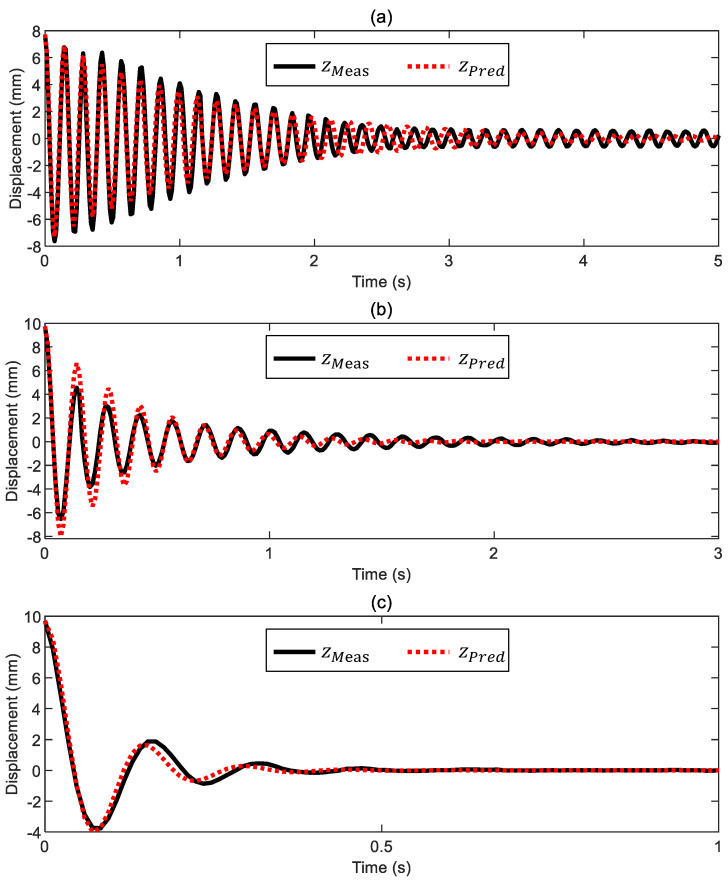
Measured ($z_{Meas}$) and simulated ($z_{Pred}$) trends of the damped free oscillations in (**a**) air, (**b**) water, and (**c**) glycol. The ball is in contact with the electromagnet surface at the beginning of the tests and is released with a step command that removes the applied voltage at t= 0 s. The plots use a different time scale for better visualization.

As anticipated in Section 2.1.1, the viscous friction force acting on the ball is assumed to be simply proportional to the ball velocity, as set out in Equation (4). Despite its simplicity, this viscous friction model is widely used in engineering to model viscous damping in single-degree-of-freedom systems, and usually is adequate to describe the damping force acting on a body that is moving at a moderate velocity in a fluid. The critical aspect of the friction model in Equation (4) is choosing a value of the viscous damping coefficient b representative of the system being considered. This step was accomplished with a set of dedicated preliminary experiments. Starting with the ball in equilibrium under its own weight, the electromagnet was powered to pull the ball down into contact with the electromagnet surface. Then, the electromagnet was unpowered with a step command to release the ball, which would then return to its original equilibrium position through a damped free oscillation. These preliminary experiments were carried out with the ball moving in air, water, and glycol. In each case, the value of the viscous damping coefficient b was empirically deduced from the observed motion, leading to a difference of an order of magnitude for the viscous damping coefficients in the different fluids ($b_{air}=$ 0.0045 kg/s, $b_{water}=$ 0.02 kg/s, and $b_{glycol}=$ 0.095 kg/s). The measured damped free oscillations of the ball in air, water, and glycol are provided in Figure 6, together with the corresponding free oscillations predicted from integrating the equation of motion, Equation (1), where the viscous friction force was modeled as explained above. As noted, the agreement between measurements and predictions is good, indicating that the simple viscous friction model in Equation (4) adequately describes the present system.

### 2.5. Closed-Loop Position Control

**Figure 7:**
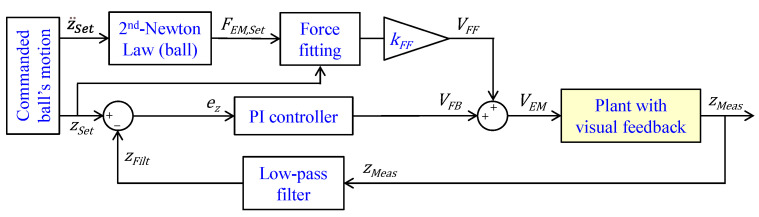
Block diagram of the proposed closed-loop position control algorithm used to generate the voltage applied to the electromagnet.

Rather than optimizing position tracking, the paper’s target is proving that our electromagnetic set-up behaves as desired even without using advanced control algorithms such as those for levitation recalled in the introduction [12,17,23,25]. Thus, we have deliberately kept the control design simple. Our controller generates a reference voltage signal for the electromagnet to track the commanded position of the ball $z_{Set}$. The control logic emerges from the block diagram shown in Figure 7. 

A feedforward term estimates the required electromagnet’s voltage $V_{FF}$ by leveraging the force equilibrium of the ball depicted in Equation (1). We do not consider the dynamics of the electromagnetic subsystem given in Equation (5) because we have experimentally verified that they are negligible (the electromagnet time scale is on the order of 10^−3^ s). The commanded acceleration ${\ddot{z}}_{Set}$ is used to calculate the desired electromagnetic force $F_{EM,Set}$. This force and the commanded ball’s position enter the force-fitting of the electromagnet to calculate the voltage command; this fitting has been rewritten accordingly in the form $V=f_{2}\left(z,\;F_{EM}\right)$ since Equation (7) is invertible:

(8) $$V=\left[\sqrt{{\left(0.2095\right)}^{2}+8.728\;F_{EM}{\left(\frac{z}{D_{b}}\right)}^{2.364}{\left(M_{b}\;g\right)}^{-1}}-0.2095\right]\left(\frac{V_{max}}{4.364}\right)$$

The dimensionless tuning term $k_{FF}$ compensates for the uncertainties of this prediction (e.g., the small error introduced in the measuring/mapping process of the electromagnetic force). For tests addressing very different operating conditions (e.g., a sinusoidal input command with variable frequency), instead of keeping $k_{FF}\;$ constant, we adjust it over time t according to the following linear trend to improve position tracking:

(9) $$k_{FF}=k_{FF,0}-k_{FF}^{*}\;t,$$

where $k_{FF,0}\;$ is the initial value and $k_{FF}^{*}$ is the rate of change. The resulting open-loop command $V_{FF}$ is then corrected by summing up the feedback term $V_{FB}$ to obtain the final voltage command $V_{EM}$ to be applied to the electromagnet. The proportional-integrative controller with gains $k_{P}$ and $k_{I}$, respectively, manipulates the filtered position error $e_{z}=z_{Set}-z_{Filt}$ resulting from the difference between the commanded position $z_{Set}$ and the filtered measured position $z_{Filt}$ and generates a corresponding voltage command $V_{FB}$. We use the filtered signal $z_{Filt}$ of the measured position $z_{Meas}$ (i.e., we apply a low-pass filter with unitary gain and cut-off frequency $\omega_{x}$ to the measured signal $z_{Meas}$). This approach removes undesired fluctuations of the measured signal from the feedback loop, especially when driving the ball inside low-viscosity fluids (air). It is, in fact, crucial to supply a smooth voltage command $V_{EM}$; otherwise, the ball’s motion might become unstable. Alternative set-ups where the ball is immersed in high-viscosity liquids allow for a more aggressive setting of the control parameters (i.e., higher values of $k_{P}$, $k_{I}$, and $\omega_{x}$) that benefit the ball’s position tracking. The exact values of these parameters will be specified later depending on the experiment being performed.

## 3. Experiments

We performed three experiments by immersing the actuated steel ball in fluids kept at room temperature but with very different viscosity μ, namely air ($\mu_{air}=$ 0.00001813 kg/ms), water ($\mu_{water}=$ 0.001002 kg/ms), and glycol ($\mu_{glycol}=$ 0.018376 kg/ms). For each scenario, we conducted an initial test commanding a sinusoidal motion profile with constant frequency; this test explores the maximum tracking performance of the current set-up. Additionally, we performed a second test to characterize the system robustness, where we imposed a sinusoidal motion profile with variable frequency.

### 3.1. Case 1: Motion in Air

**Figure 8:**
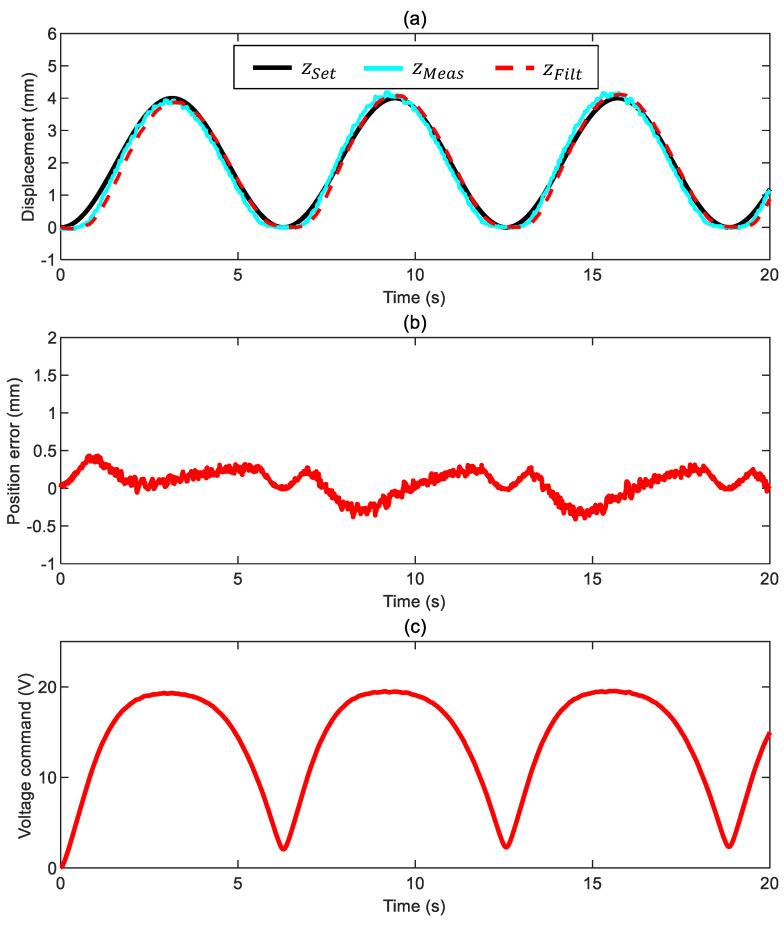
Closed-loop motion control of the ball surrounded by air with a constant-frequency position command (Video S1 and control parameters $k_{FF}=$ 1, $k_{P}=$ 2.2 V/mm, $k_{I}=$ 1.8 V/mm/s, and $\omega_{x}=$ 7 Hz): (**a**) ball position (commanded position $z_{Set}$, measured position $z_{Meas}$, and filtered measured position $z_{Filt}$), (**b**) position error $e_{z}^{*}=z_{Set}-z_{Meas}$, and (**c**) voltage command.

As mentioned, we start by exploring the maximum tracking potential of the proposed set-up. We applied a sinusoidal position command with a constant frequency of 0.16 Hz to move the ball 4 mm in the z direction below its equilibrium point. The position tracking in Figure 8a has a minimum overshoot (no more than 0.25 mm) and negligible phase delay. Figure 8b shows that the actual tracking error $e_{z}^{*}=z_{Set}-z_{Meas}$, calculated with the measured position, stays within ±0.5 mm. Some fluctuations characterize the trend of such a position error; for this reason, we introduced in the feedback loop the filtered error $e_{z}$ obtained with the filtered position signal. It is also worth noticing that the electromagnetic force is permanently active (Figure 8c depicts that there is always a nonzero voltage command); this is the driving force when lowering the ball from its equilibrium point. Conversely, the electromagnetic force switches its role during the lifting stage since it controls the ball position by counteracting the driving force of the elastic spring. This dual behavior explains the different tracking performance between the lowering and lifting stages. The ball motion is always very smooth, which is a remarkable outcome due to the almost negligible drag friction of this configuration.

It is worth noticing that the ball motion is 1D since it moves vertically inside the tube without touching it (the videos in the attached material support this statement). This behavior occurs because we have correctly selected the control gains. An improper gain selection (i.e., too aggressive settings) causes loss of control with an unacceptable swinging oscillation of the ball (such a scenario was confirmed by experiments not reported herein).

**Figure 9:**
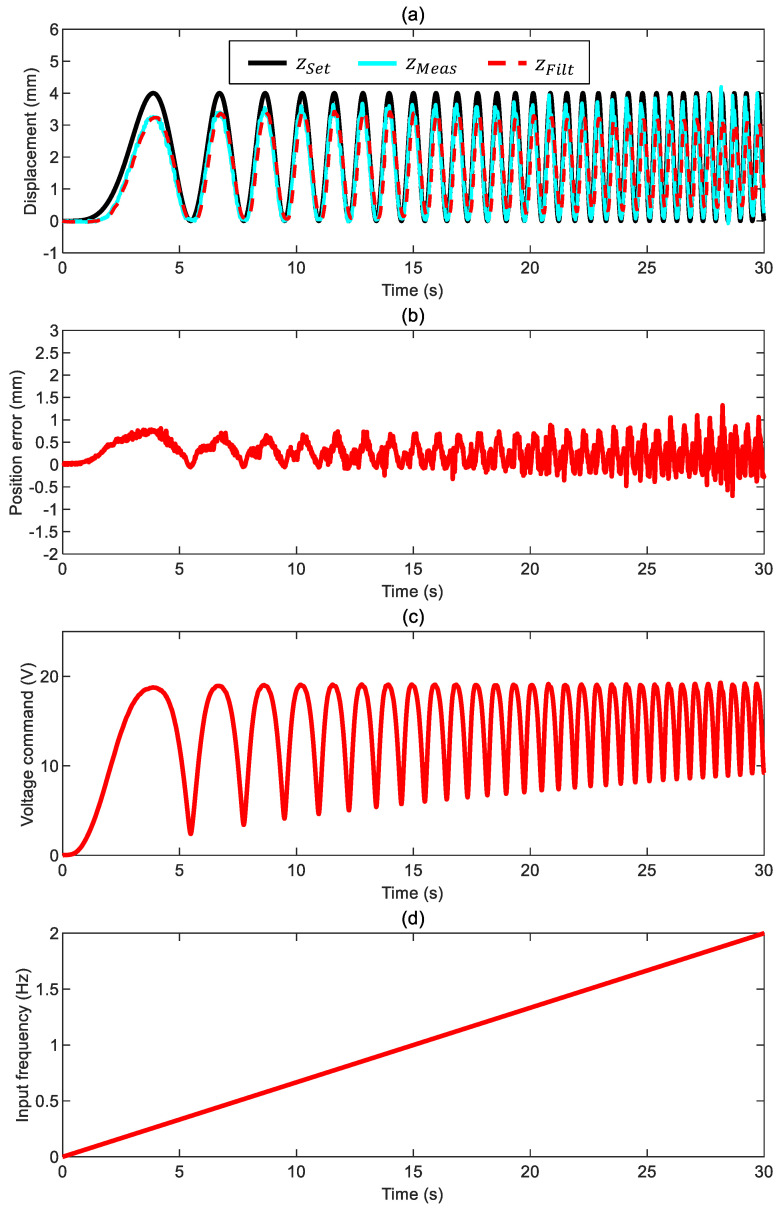
Closed-loop control of the ball in air with a variable-frequency command (Video S2 and control parameters $k_{FF,0}=$ 1, $k_{FF}^{*}=$ 0.05/30 1/s, $k_{P}=$ 1.5 V/mm, $k_{I}=$ 1.05 V/mm/s, and $\omega_{x}=$ 15 Hz): (**a**) ball position (commanded position $z_{Set}$, measured position $z_{Meas}$, and filtered measured position $z_{Filt}$), (**b**) position error $e_{z}^{*}=z_{Set}-z_{Meas}$, (**c**) voltage command, and (**d**) input frequency.

Considering a more challenging scenario, we performed a second test where the frequency of the sinusoidal position command varies linearly up to 2 Hz. Figure 9 depicts the results confirming that the system can keep up while providing a smooth response characterized by a voltage command that does not saturate. The risk is again losing the ball control because there is almost no damping in this scenario; this unfortunate event is not the case even if the actual position error $e_{z}^{*}$ deteriorates progressively even if it stays within ±1 mm. The phase delay is generally negligible, while undershooting characterizes the position error. Again, there is a slightly asymmetric system response between the lowering and lifting stages, where the latter scenario results are more accurate.

### 3.2. Case 2: Motion in Water

**Figure 10:**
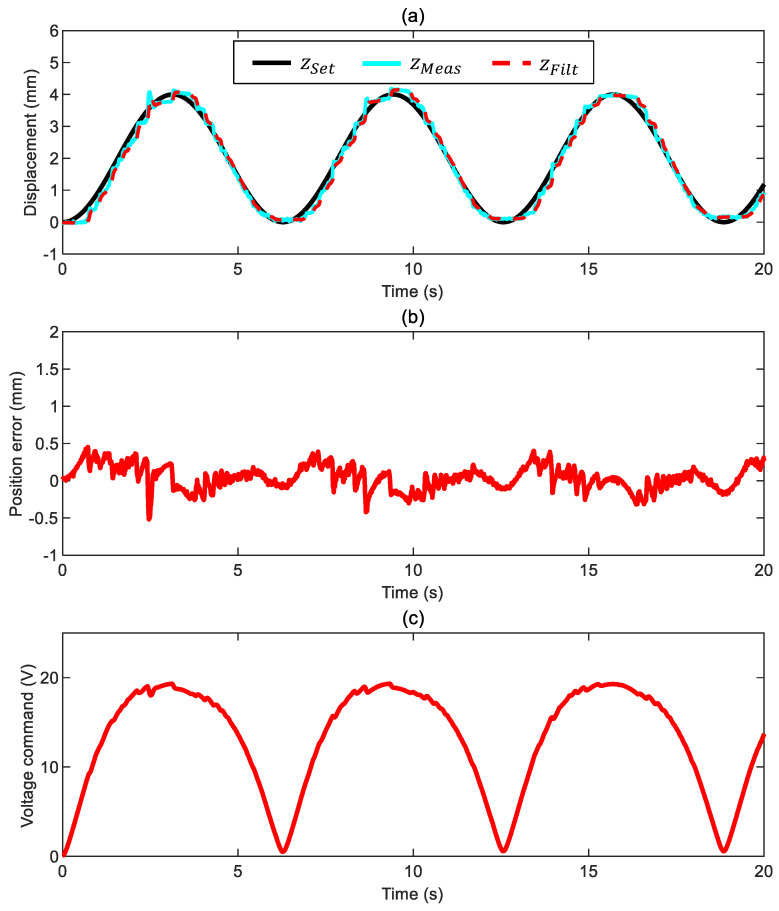
Closed-loop motion control of the ball surrounded by water with a constant-frequency command (Video S3 and control parameters $k_{FF}=$ 1, $k_{P}=$ 1.8 V/mm, $k_{I}=$ 1.2 V/mm/s, and $\omega_{x}=$ 25 Hz): (**a**) ball position (commanded position $z_{Set}$, measured position $z_{Meas}$, and filtered measured position $z_{Filt}$), (**b**) position error $e_{z}^{*}=z_{Set}-z_{Meas}$, and (**c**) voltage command.

When repeating the same constant-frequency test described in the previous section while submerging the ball in water, the tracking performance is aligned to the scenario in air with a position error that stays within ±0.5 mm (Figure 10). We used a low-pass filter with a much higher cut-off frequency, 25 Hz, compared to 7 Hz for the case in air. In practical terms, we can apply a feedback command containing some fluctuations since water’s higher viscosity introduces a friction term that damps out undesired oscillations. The voltage command again varies smoothly. 

**Figure 11:**
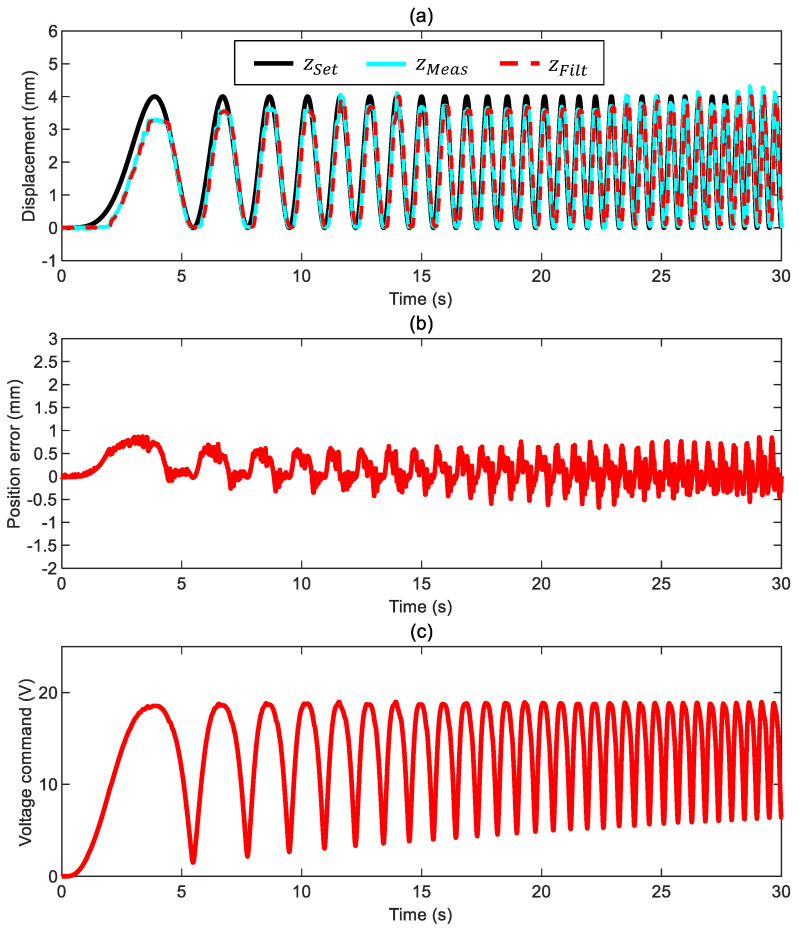
Closed-loop control of the ball in water with a variable-frequency position command (Video S4 and control parameters $k_{FF,0}=$ 1, $k_{FF}^{*}=$ 0.005 1/s, $k_{P}=$ 1.05 V/mm, $k_{I}=$ 0.7 V/mm/s, and $\omega_{x}=$ 30 Hz): (**a**) ball position (commanded position $z_{Set}$, measured position $z_{Meas}$, and filtered measured position $z_{Filt}$), (**b**) position error $e_{z}^{*}=z_{Set}-z_{Meas}$, (**c**) and voltage command.

Moving to the scenario with a variable-frequency (up to 2 Hz) sinusoidal command reported in Figure 11, the results show that the position tracking has an actual error $e_{z}^{*}$ well within ±1 mm with a minimum phase delay. The system response is smooth enough even if the low-pass filter’s cut-off frequency was doubled compared to the case in air (30 Hz compared to 15 Hz).

### 3.3. Case 3: Motion in Glycol

**Figure 12:**
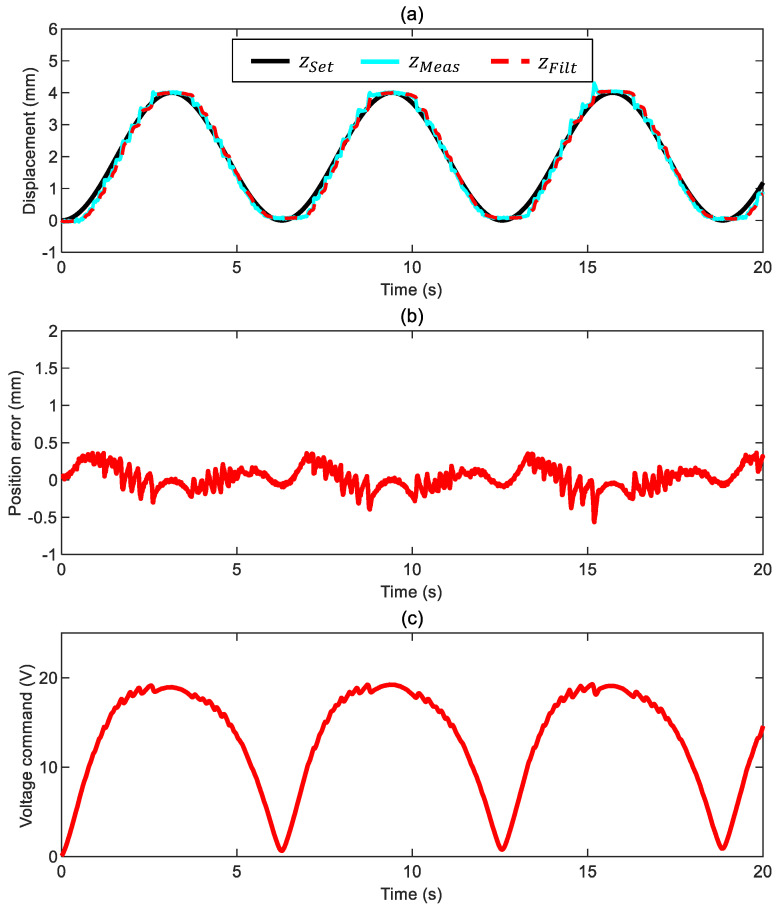
Closed-loop control of the ball in glycol with a constant-frequency position command (Video S5 and control parameters $k_{FF}=$ 1, $k_{P}=$ 3 V/mm, $k_{I}=$ 2.5 V/mm/s, and $\omega_{x}=$ 25 Hz): (**a**) ball position (commanded position $z_{Set}$, measured position $z_{Meas}$, and filtered measured position $z_{Filt}$), (**b**) position error $e_{z}^{*}=z_{Set}-z_{Meas}$, and (**c**) voltage command.

**Figure 13:**
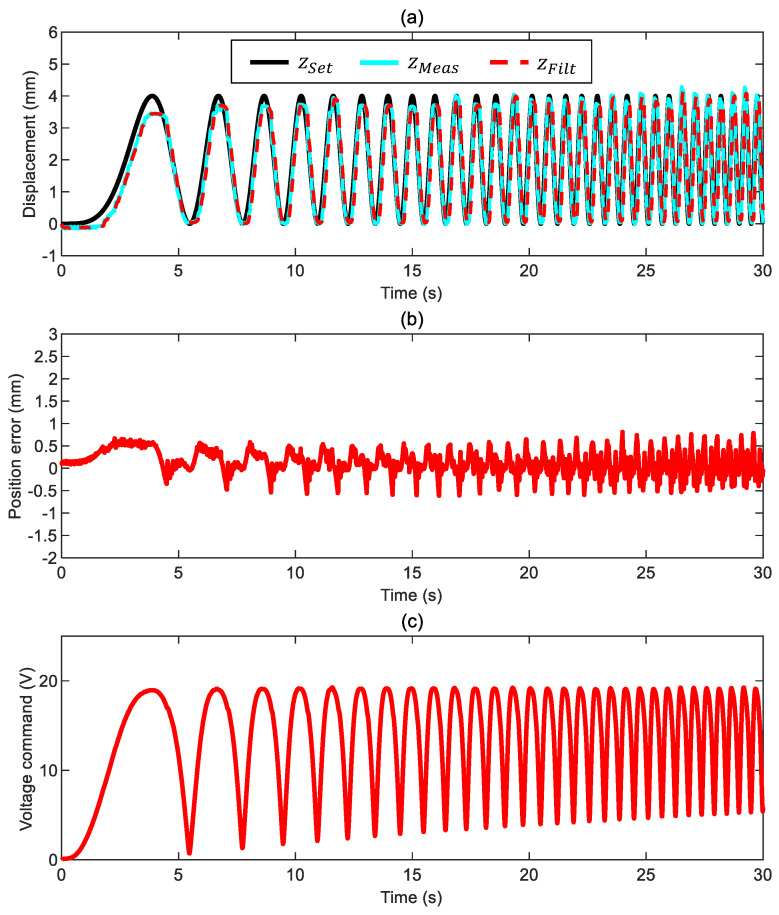
Closed-loop motion control of the ball in glycol with a variable-frequency position command (Video S6 and control parameters $k_{FF,0}=$ 1, $k_{FF}^{*}=$ 0.1/30 1/s, $k_{P}=$ 0.8 V/mm, $k_{I}=$ 0.7 V/mm/s, and $\omega_{x}=$ 30 Hz): (**a**) ball position (commanded position $z_{Set}$, measured position $z_{Meas}$, and filtered measured position $z_{Filt}$), (**b**) position error $e_{z}^{*}=z_{Set}-z_{Meas}$, (**c**) voltage command.

The results of the tests with the ball immersed in glycol (Figure 12 and Figure 13) resemble the ones in water. The actual position error stays within ±0.5 mm, the phase delay is always negligible, and the commanded voltage exhibits a smooth trend despite the higher cut-off frequency used to filter the measured position. It becomes apparent that increasing the system’s viscous friction above a certain level (see the case in water) brings marginal improvement to the overall system response.

### 3.4. System Comparison

After discussing the experimental results individually, we compare them in this section. We start by providing an overview of the control parameters selected for our experiments (Table 1). It is apparent that low-viscosity fluids, such as air, require very smooth input commands to avoid unwanted oscillations; filtering the measured position with a low cut-off frequency is the most effective way to enforce this situation. Once a relatively smooth signal is fed back, the settings of the proportional and integrative gains play a minor role in the system response.

**Figure 14:**
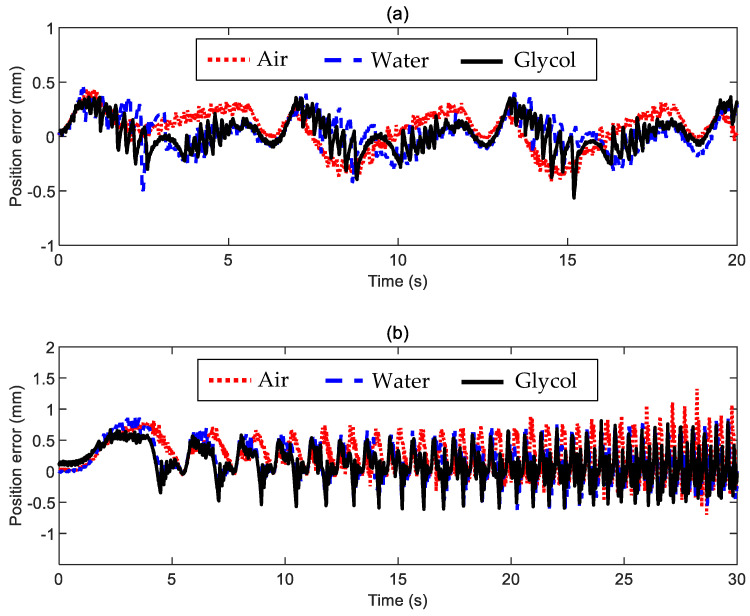
Comparison of the actual position error $e_{z}^{*}=z_{Set}-z_{Meas}\;$ resulting from the tests reported above and performed in different conditions (air, water, and glycol): (**a**) constant-frequency position command, and (**b**) variable-frequency position command.

In this regard, Figure 14a refers to the experiments with a constant-frequency position command. It shows that the proposed set-up can maintain a consistent performance when proper control settings are chosen even if the environmental conditions change drastically; we are comparing cases with almost no friction (motion in air), moderate friction (motion in water), and significant friction (motion in glycol). The tracking error calculated with the measured position is almost indistinguishable in terms of both accuracy (it remains well within ±0.5 mm, with negligible phase delay) and fluctuations (the input command is always very smooth). Moreover, Figure 14b recalls the experiments with a variable-frequency position command (i.e., the selection of the control settings must be a compromise to deal with very different dynamic scenarios sufficiently well). Again, the system performance is consistent among the three cases. The actual position error remains approximately within ±1 mm for the instances in air and water. It is, however, apparent that the high viscosity of glycol helps improve the position tracking at higher frequencies.

Finally, Table 2 lists the root mean square (RMS) values of the same errors plotted in Figure 14. The RMS value indicates, on average, how far the error is from zero. Thus, an increased viscous friction benefits the average system behavior. When considering constant-frequency commands, the RMS values of the position error in water and glycol become 85.6% and 79.1%, respectively, with respect to the case in the air. Moving to the tests with variable-frequency commands, the RMS values of the position error in water and glycol become 90% and 75.1% concerning the case in the air.

## 4. Conclusions

In this research paper, we have developed a novel and integrated methodology for applying electromagnetic actuation for closed-loop position control using visual feedback. The main contributions of our study are summarized as follows:

1. We have proposed a user-friendly set-up that relies on cost-effective, off-the-shelf components. This set-up combines three main subsystems: actuation, motion tracking, and feedback control. Our approach has minimum complexity since we have chosen a plug-and-play webcam for optical tracking and developed an intuitive, model-based control law.
2. We have gained significant insight into the distribution of the magnetic flux/magnetic force of electromagnets. We have shown that the magnetic flux is highly nonlinear and severely changes when a ferromagnetic object is nearby. We have also done an accurate and experimentally validated fitting of the electromagnetic force by creating an invertible function to be used for the control design.
3. We have used an off-the-shelf webcam to implement motion tracking via visual feedback. The camera samples images in the range of 85 ± 2 fps. Our conservative estimate for the sensing accuracy of the ball centroid’s location is about ±300 microns. We have validated this approach with an oscillating pendulum whose power spectrum presents one dominant peak that closely matches its theoretical oscillation frequency.
4. We have deliberately designed a control algorithm for closed-loop, position control of the actuated object with minimum complexity. This law combines a model-based feedforward term (it predicts the electromagnet’s voltage command based on the desired motion) and a feedback term (it corrects the voltage command by applying proportional-integrative control on the position error).
5. We have performed closed-loop position tracking (1D vertical motion) with a 10 mm steel ball hanging from a low-stiffness spring and surrounded by diverse fluids (air, water, and glycol). Despite the different conditions, our set-up can consistently perform well when proper control settings are chosen. When commanded by a sinusoidal position with constant frequency, the tracking error stays within ±0.5 mm with a negligible phase delay.

The present set-up for electromagnetic actuation performs well at a relatively low actuation frequency. Resorting to a higher spec machine-vision set-up and migrating from the general-purpose MATLAB-Simulink computing environment to a dedicated machine-vision software are possibilities to overcome the present frequency-related limitations. These approaches will be explored in future investigations. In addition, the low-cost characteristics and straightforward structure make this integrated methodology an excellent candidate for future research. There are some areas that deserve further investigation, such as designing electromagnets with more suitable trends of the force characteristics. The proposed approach is of interest for industry-relevant applications. While maintaining a single-electromagnet set-up, the natural extension addresses levitation since it gives room for developing advanced control algorithms. Additionally, a system with two or more electromagnets has already been envisioned to control the 2D planar motion of objects, including ferrofluids.

## Figures and Tables

**Table 1:** Synthesis of the control settings used to perform our experimental validations.

Test	$k_{FF,0}$(-)	$k_{FF}^{*}$(-)	$k_{P}$(V/mm)	$k_{I}$(V/mm/s)	$\omega_{x}$(Hz)
Air (constant frequency)	1	0	2.2	1.8	7
Air (variable frequency)	1	0.05/30	1.5	1.05	15
Water (constant frequency)	1	0	1.8	1.2	25
Water (variable frequency)	1	0.005	1.05	0.7	30
Glycol (constant frequency)	1	0	3	2.5	25
Glycol (variable frequency)	1	0.1/30	0.8	0.7	30

**Table 2:** RMS values in mm of the actual position error $e_{z}^{*}=z_{Set}-z_{Meas}\;$ resulting from the tests reported above and performed in different conditions (air, water, and glycol).

Test	RMS Value	Test	RMS Value
Air (constant frequency)	0.1884	Air (variable frequency)	0.3857
Water (constant frequency)	0.1612	Water (variable frequency)	0.3471
Glycol (constant frequency)	0.1491	Glycol (variable frequency)	0.2896

## Data Availability

The data presented in this study are available upon request from the corresponding authors.

## Supplementary Materials

The following supporting information can be downloaded at https://www.mdpi.com/article/10.3390/s24092760/s1. The following videos are displayed in real-time (1×): Video S1: Closed-loop motion control of the ball surrounded by air with a constant-frequency position command. Video S2: Closed-loop motion control of the ball surrounded by air with a variable-frequency position command. Video S3: Closed-loop motion control of the ball surrounded by water with a constant-frequency position command. Video S4: Closed-loop motion control of the ball surrounded by water with a variable-frequency position command. Video S5: Closed-loop motion control of the ball surrounded by glycol with a constant-frequency position command. Video S6: Closed-loop motion control of the ball surrounded by glycol with a variable-frequency position command.
